# Supplementary figures and images for: The COVID-19 pandemic “anthropause” decreased plastic ingestion in neotropic cormorants Nannopterum brasilianus in Lima, Peru
Source: PeerJ. 2024 May 30;12:e17407. doi: 10.7717/peerj.17407 (PMC11144399; doi:10.7717/peerj.17407)

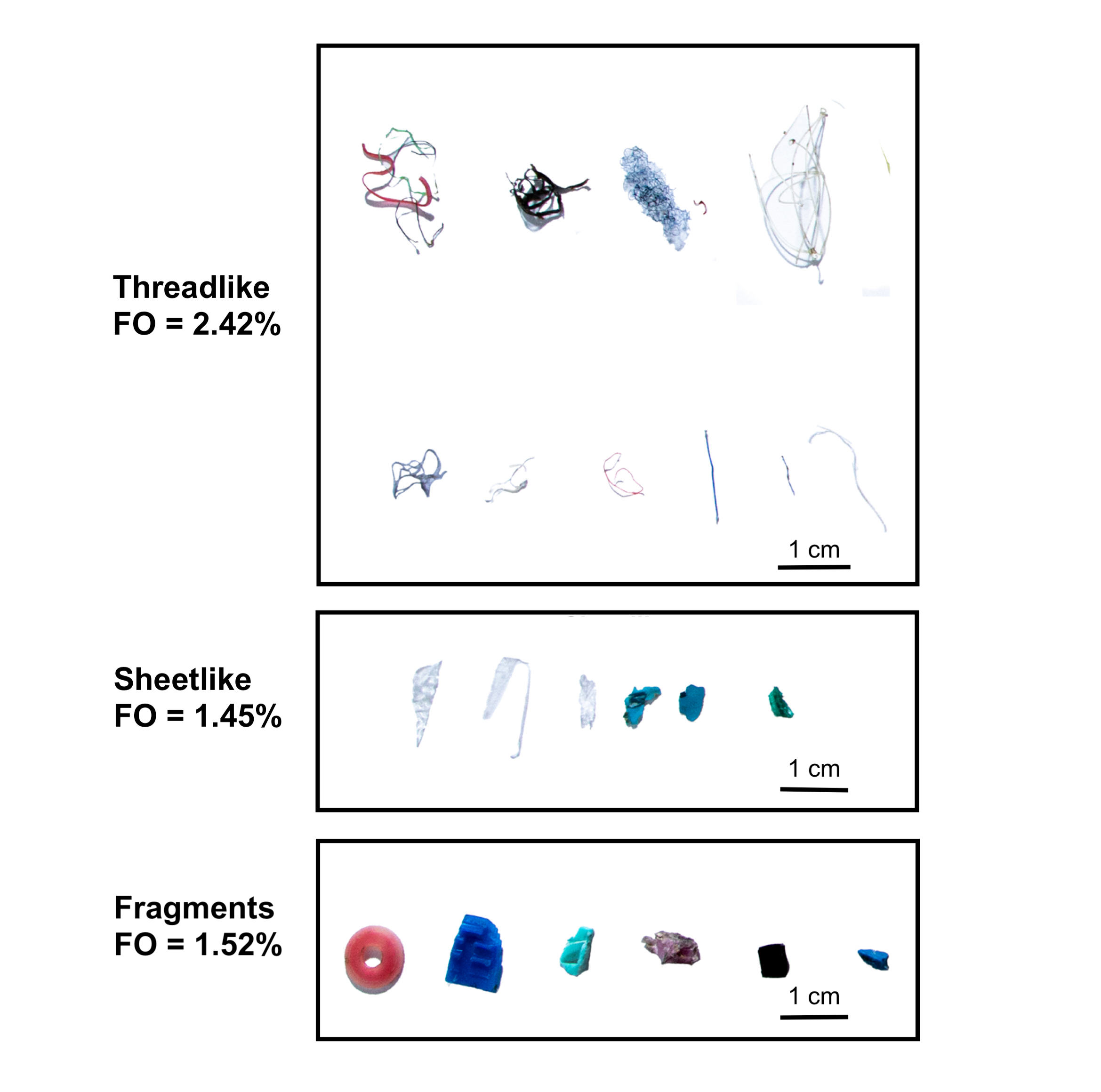

Supplement: Supplemental Information 1 [file peerj-12-17407-s001.png]

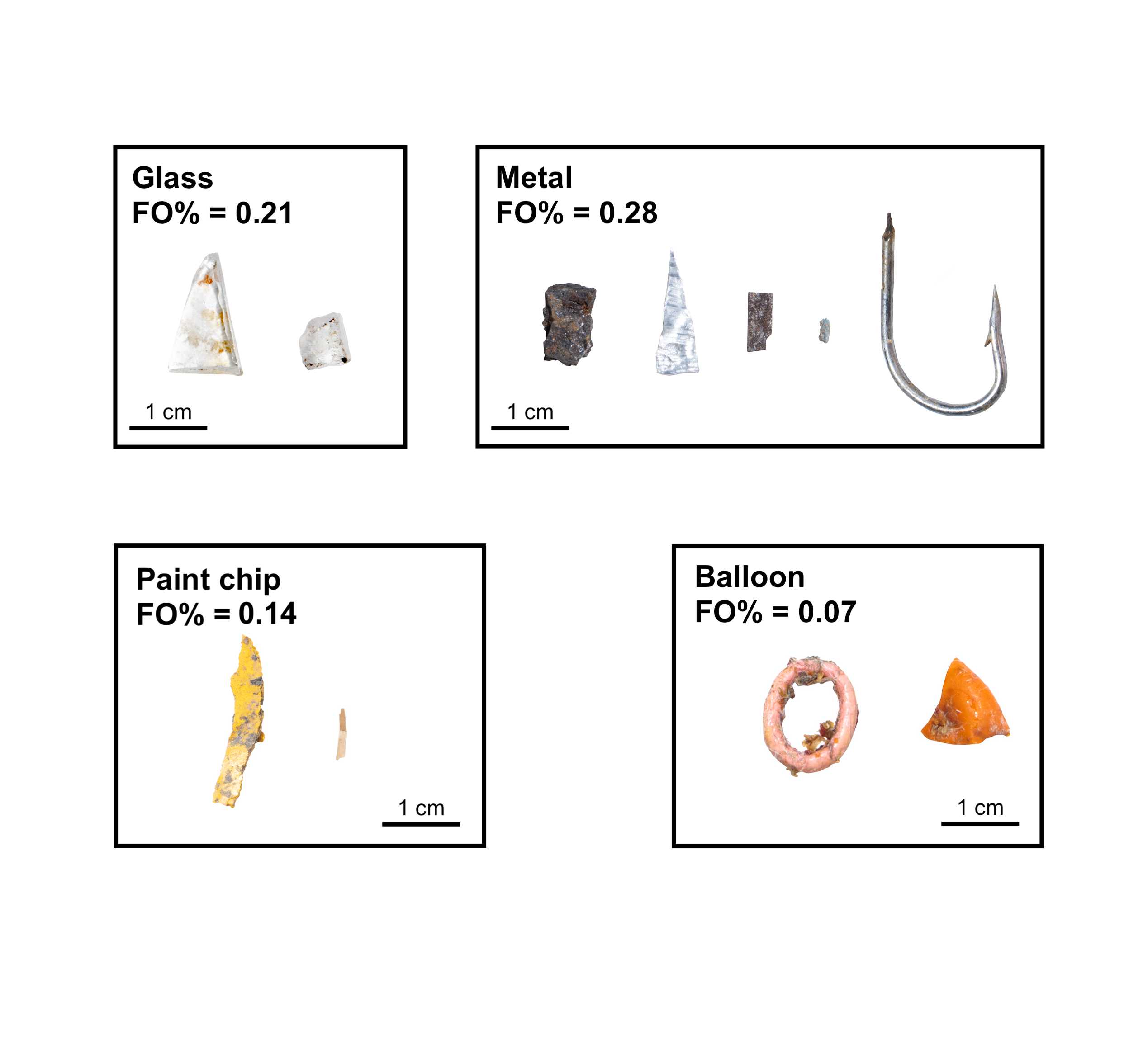

Supplement: Supplemental Information 2 [file peerj-12-17407-s002.png]

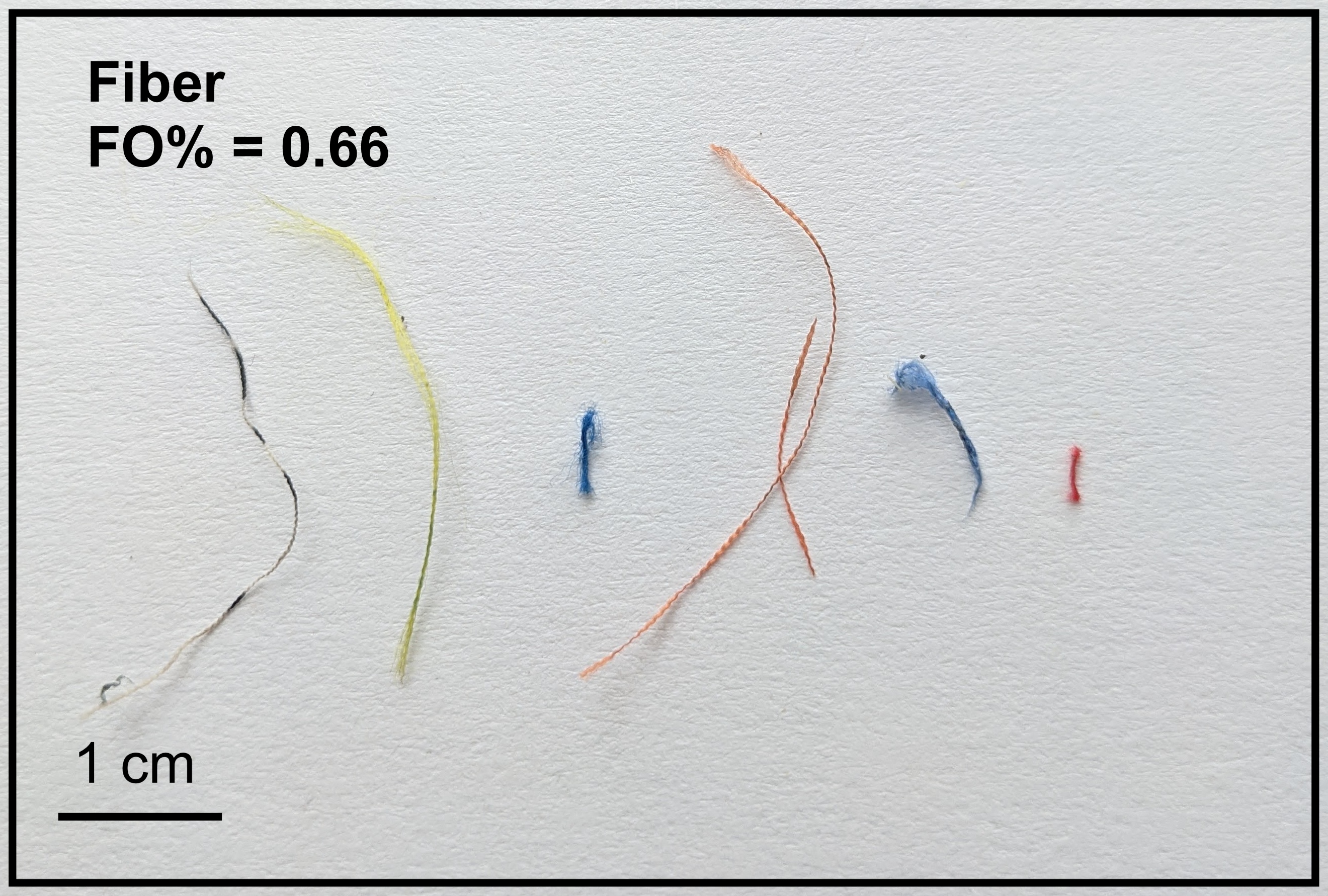

Supplement: Supplemental Information 3 [file peerj-12-17407-s003.jpg]
