# Supplementary material for: The COVID-19 pandemic “anthropause” decreased plastic ingestion in neotropic cormorants Nannopterum brasilianus in Lima, Peru
Source: PeerJ. 2024 May 30;12:e17407. doi: 10.7717/peerj.17407 (PMC11144399; doi:10.7717/peerj.17407)
Supplement: Supplemental Information 7 [file peerj-12-17407-s007.docx]

Subject: Rebuttal Letter for the addition of a new author. MS The COVID-19 pandemic “anthropause” decreased plastic ingestion in Neotropic cormorants *Nannopterum brasilianus* in Lima, Peru.

Dear Editor:

We would like to express our gratitude for the thorough review and constructive feedback provided on our manuscript titled " The COVID-19 pandemic “anthropause” decreased plastic ingestion in Neotropic cormorants *Nannopterum brasilianus* in Lima, Peru:" We appreciate the opportunity to address the concerns raised by the reviewers, particularly regarding the lack of solid arguments to support our findings on neotropic cormorants' plastic ingestion patterns during the pandemic compared to pre-pandemic conditions.

One of the reviewers rightly highlighted the possibility that differences observed between phases could be attributed to shifts in prey consumption, assuming that different prey may contain varying amounts of plastic in their bodies. In response to this concern, we thoroughly analyzed the diet composition of neotropic cormorants before and during the pandemic periods. The pellets analyzed in our study underwent meticulous dissection, and all items, including plastic and fish otoliths, were sorted and counted. While our initial manuscript focused primarily on anthropogenic debris, we have now included the results of diet composition to address this important aspect raised by the reviewer.

It is important to note that all diet composition data presented in our revised manuscript were part of another study conducted on the diet composition of neotropic cormorants in two localities, including the study area of our manuscript. The dissection, sorting, otolith identification, and analysis were undertaken by Alvaro Rios, a valued member of our lab team. In recognition of his substantial contribution to this aspect of the research, we have included him as a co-author of the revised manuscript where the diet composition data is presented.

We believe that the inclusion of these additional findings strengthens our manuscript and provides a more comprehensive understanding of the factors influencing neotropic cormorants' plastic ingestion patterns. We are confident that these revisions address the concerns raised by the reviewers and enhance the overall quality of our manuscript.

Thank you once again for your time and consideration.

Sincerely,

Carlos Zavalaga

Director Unidad de Investigacion de Ecosistemas Marinos

Universidad Científica del Sur
